# Effectiveness of an Autologous Micrografting Technology for Treating Stretch Marks

**DOI:** 10.1111/jocd.70321

**Published:** 2025-06-30

**Authors:** Andrea Garelli, Valeria Pessei, Ruggero Tagliabue, Olha Sles, Ratchathorn Panchaprateep

**Affiliations:** ^1^ Department of Systems Medicine University Tor Vergata Rome Italy; ^2^ Department Chemistry, Biology, Biotechnology University of Perugia Perugia Italy; ^3^ SHRO Italia Foundation ETS Candiolo (TO) Italy; ^4^ Exel Poliambulatorio Monza Italy; ^5^ Lazerhous Clinic Kiev Ukraine; ^6^ Division of Dermatology, Department, Faculty of Medicine Chulalongkorn University Bangkok Thailand; ^7^ Absolute Hair Clinic Bangkok Thailand

**Keywords:** autologous micrografts, collagen, elastin, gene expression, stretch marks

## Abstract

**Background:**

Stretch marks or striae distensae (SD) are common dermal lesions caused by the disruption of collagen and elastin fibers in the skin, often triggered by rapid mechanical stretching. Despite the availability of numerous treatment modalities, from topical agents to energy‐based devices, no single therapy has demonstrated consistent, long‐term efficacy across all patient populations. The pathophysiology of SD involves complex alterations in the extracellular matrix (ECM), particularly affecting fibroblast activity and collagen/elastin synthesis.

**Aims:**

This pilot study aims to evaluate the clinical and molecular efficacy of autologous micrografting technology as a novel therapeutic option for SD. Specifically, it investigates the treatment's impact on ECM‐related gene expression and overall skin appearance.

**Patients/Methods:**

Fourteen patients (13 females, 1 male) with clinically evident SD were enrolled. All participants underwent a standardized treatment protocol comprising microneedling followed by intradermal injection of autologous micrografts, obtained via a minimally invasive procedure. Clinical assessments were performed through standardized photography at baseline, 1 month, and 6 months post‐treatment. In vitro assays were conducted on cultured human dermal fibroblasts exposed to the micrograft suspension.

**Results:**

Clinical evaluations showed noticeable aesthetic improvements, including reduced striae visibility and improved skin texture, with high patient‐reported satisfaction. Molecular analyses revealed the upregulation of key ECM genes, including COL4A1, COL6A1, and ELN, indicating enhanced fibroblast activation and regenerative potential.

**Conclusions:**

Autologous micrografting appears to be a promising, biologically active approach for SD treatment. It promotes ECM remodeling by stimulating fibroblast function and may represent a valuable addition to the therapeutic landscape for stretch marks.

## 1 Introduction

Stretch marks, medically also known as *striae distensae* (SD), are a type of dermal scarring characterized by linear streaks on the skin and can be classified into two main types based on their appearance and stage of development [1]. *Striae rubrae* (SR) are the initial stage, appearing as red, pink, or purple marks due to vascular dilation, which makes them more responsive to treatments. Over time, they evolve into *striae albae* (SA), which are mature stretch marks that appear white or silvery and are more challenging to treat due to reduced vascularity and collagen density [2]. Usually, stretch marks can occur due to the tearing of the dermis, often as a result of rapid skin stretching associated with growth spurts, weight gain, pregnancy, or hormonal changes [3, 4]. These marks commonly develop on areas prone to significant stretching, such as the abdomen, thighs, buttocks, breasts, and upper arms, but can occur anywhere on the body [5, 6]. Previous research studies indicate that between 11% and 88% of women develop stretch marks on some part of their body, with postpartum prevalence [7, 8]. In healthy skin, collagen bands in the upper reticular dermis are typically elongated and aligned parallel to the skin surface, but this structure is disrupted in areas affected by striae that present a rupture and separation of collagen fibers [9, 10].

Electron microscopy reveals that the early changes in SD are marked by mast cell degranulation and macrophage activation, leading to moderate dermal elastolysis. This suggests that mast cells, through the production and release of elastases, play a critical role in triggering the pathogenesis of SD [11]. Structural changes in collagen fibers and fibroblast abnormalities become evident as SD progresses to the clinically visible stage; at this point, mast cells diminish, while dermal edema increases, accompanied by lymphocyte infiltration [12]. The initial phase of SR is defined by inflammatory lesions, whereas the SA phase is characterized by epidermal atrophy and collagen disruptions resembling dermal scar tissue under the microscope [13]. Despite numerous studies investigating SD's etiology, there is considerable variation in findings, and no universally accepted treatments are currently available [2].

Collagen and elastin are fundamental structural proteins that contribute to the mechanical properties of the skin, including its strength, elasticity, and resilience. The genes encoding these proteins, primarily COL1A1, COL1A2 (for type I Collagen), COL3A1 (for type III Collagen), and ELN (for Elastin), play crucial roles in the skin remodeling process, particularly in response to mechanical stress, aging, and injury [14, 15].

Skin remodeling involves a dynamic balance between extracellular matrix (ECM) synthesis and degradation, mediated by fibroblasts and matrix metalloproteinases (MMPs). Specifically, collagen provides tensile strength, while elastin ensures the skin's ability to return to its original shape after stretching [16, 17]. The dysregulation in collagen and elastin gene expression can impair these processes, contributing to dermatological conditions such as stretch marks. In addition, histological studies reveal a reduction in collagen and elastin fibers, along with alterations in COL1A1, COL3A1, and ELN gene expression, leading to ECM disorganization and reduced skin elasticity [2, 18]. For this reason, understanding these molecular mechanisms is essential for developing targeted therapies aimed at enhancing collagen synthesis and restoring ECM integrity in affected individuals.

Various approaches exist, each with differing levels of effectiveness: topical treatments, such as retinoids and hyaluronic acid, aim to improve the skin's texture and stimulate collagen production but are generally more effective on early stage stretch marks (*striae rubrae*) [2]. Laser therapy, like fractional laser and pulsed dye laser targets skin pigmentation and stimulates collagen remodeling, showing promising results for both red and white marks [19]. Microneedling and radiofrequency devices work by creating micro‐injuries to induce collagen regeneration [20], while newer techniques, like autologous micrografting, leverage the body's own regenerative cells to repair damaged tissue. Additionally, chemical peels and dermabrasion are used to exfoliate and rejuvenate the skin, although their efficacy is often limited [21].

Despite advancements, no single treatment guarantees complete elimination, and results can vary based on the severity of the stretch marks, skin type and the chosen intervention.

While SD are not considered a medical condition, their visible nature can be disfiguring and lead to psychological or emotional distress for many women and men; furthermore, effective prevention and treatment options remain limited. In the last years, new innovative technologies have been introduced for the treatment of aesthetic problems such as stretch marks; among them, Rigenera Technology (Human Brain Wave Srl, Turin, Italy) has shown promising results in the treatment of SD [22, 23]. This procedure involves harvesting micrografts from the patient's own tissue, reducing the risk of adverse reactions in a minimally invasive manner.

On the basis of considerations above, the aim of this study is to evaluate the efficacy of autologous micrografting technology in the treatment of SD and their action in modulating gene expression of factors involved in skin remodeling such as collagen and elastin.

## 2 Materials and Methods

### 2.1 Study Design and Patient's Demographics Information

The present study enrolled 14 patients who were treated by three different physicians to ensure consistency and reproducibility of the procedure; the average age of the patients was 39.2, comprising 1 male and 13 females. The study was conducted over a period of 9 months, from April 2024 to January 2025, allowing for adequate follow‐up and assessment of treatment outcomes. The patients were carefully selected based on predefined inclusion criteria to evaluate the efficacy of the treatment for SD using the new Rigenera Technology protocol.

### 2.2 Eligibility Criteria

Patients eligible for inclusion must be able to provide informed consent, be healthy males or females aged between 24 and 65 years with Fitzpatrick‐Goldman skin types I–IV, and have visible SD, specifically *striae rubrae* or *striae albae*.

Participants were excluded if they had Fitzpatrick‐Goldman skin types V–VI, were pregnant, or were less than 3 months postpartum. Exclusions also applied to those with dermal or epidermal damage, recent surgery in the treated area, or hormonal disorders.

In addition, active Herpes Simplex, multiple dysplastic nevi, bleeding disorders, anticoagulant use, immunosuppression, cancer, or tattoos in the treated area also disqualified participants. Any condition deemed unsafe by the physician led to exclusion.

### 2.3 Treatment Protocol

#### 2.3.1 Pre‐Procedure

Patients should avoid non‐steroidal anti‐inflammatory drugs for at least 24 h before treatment. The procedure began with microneedling to induce initial skin damage; next, the treatment area was carefully cleaned and disinfected with 70% alcohol. A dermapen equipped with 32‐needle heads set to a depth of 1.5 mm was used, with the needles positioned perpendicularly to the stretched skin to precisely target the base of the scars. Pressure was applied systematically across all stretch mark areas until pinpoint bleeding occurred; any blood was gently removed with sterile gauze, and the skin was cleaned with sterile saline.

#### 2.3.2 Estriaephil Micrografting Procedure

The donor site was prepared with an antiseptic solution, marked, and anesthetized with 2% lidocaine. Three 2.5 mm biopsy punches were taken from the mastoid area and placed in the Rigeneracons (7945TS) grid with 6 mL of physiological saline. The samples were processed for 2 min using the Sicurdrill device, and the resulting micrograft suspension was collected into syringes. The micrograft suspension was injected intradermally into the SD at 1 cm intervals using 30G needles, creating small papules.

#### 2.3.3 Post‐Procedure

Patients should avoid washing the treated area for 24 h and refrain from applying antibiotics or exposing the area to the sun for 7 days with sunscreen protection.

### 2.4 Photographic Assessment

**FIGURE 1:**
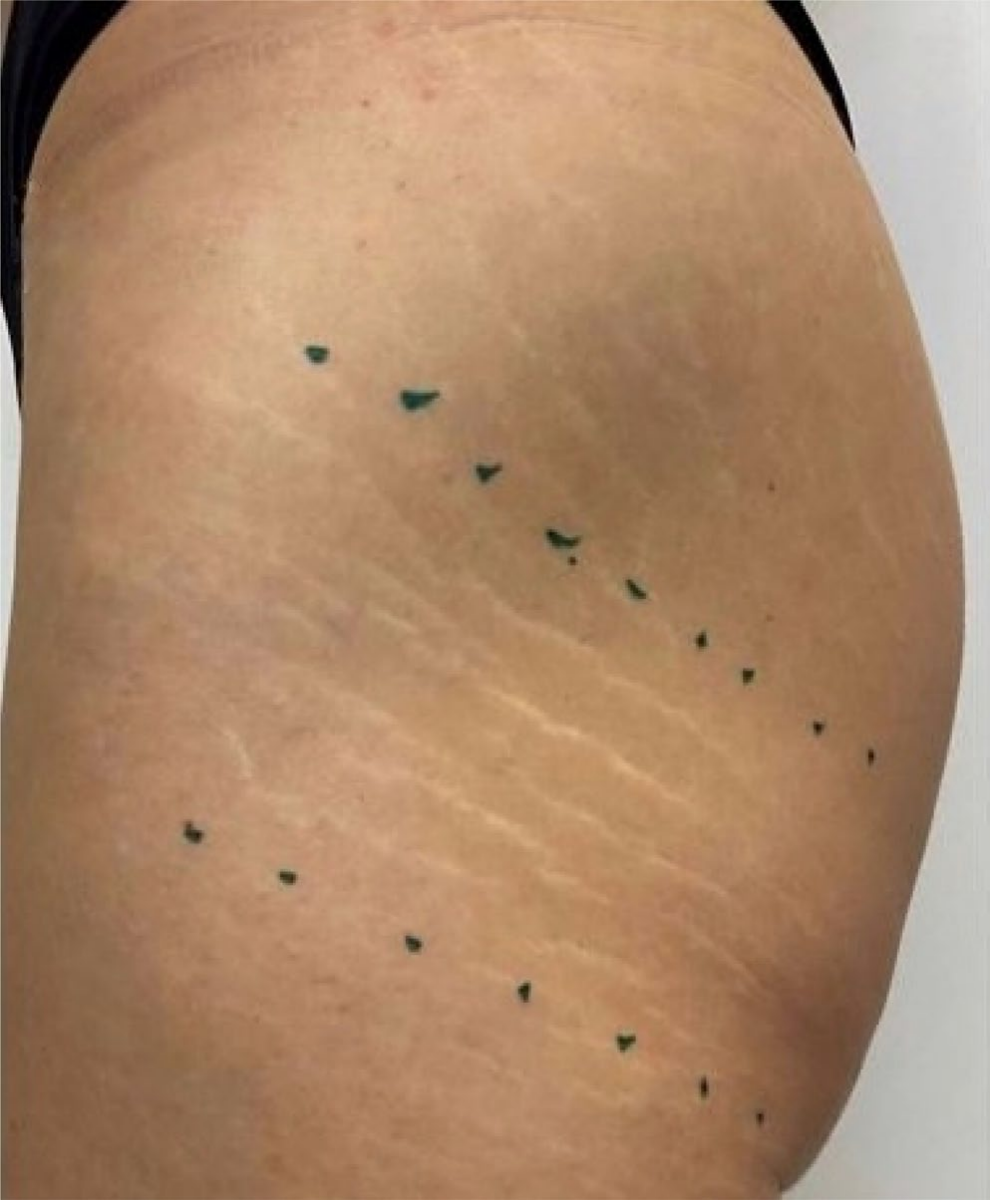
Clinical appearance of the gluteal region showing stretch marks and pre‐procedural markings. The green marks indicate reference points for the planned procedure.

The photographic assessment was conducted by physicians both before the treatment and during the follow‐up visits at 1‐ and 6‐month post procedure, for patients undergoing the protocol. To ensure consistency and reproducibility, a standardized procedure was followed: the location and position of the patient were carefully marked and replicated during each session to maintain comparability; optimal illumination was ensured using a spotlight with fixed settings such as medium light, constant intensity, and a distance of 20–40 cm from the treatment area. (Figure 1) A minimum of three photographs were taken for each session, capturing the targeted SD from consistent angles. Reference points such as moles, freckles, or birthmarks were utilized to accurately identify the same location in subsequent follow‐ups.

### 2.5 In Vitro Tests

In vitro analyses were performed to assess the difference in Collagen and Elastin gene expression between fibroblasts cultured in the micrograft suspension and in the normal medium.

Cells were seeded at a density of 1 × 10^6^ cells/mL in 25 cm^2^ flasks and incubated at 37°C for 24 h to allow cell adhesion to the flask surface. After this initial period, four different treatments were applied. For each treatment, 500 μL of the micrograft suspension was added to the culture medium, resulting in a final volume of 2 mL and a study solution concentration of 25% in the total medium. The experimental conditions were as follows:

1. A micrograft suspension generated by processing human skin biopsies with the Rigeneracons device.
2. A control condition where cells were cultured in commercial Iscove's Modified Dulbecco's Medium (IMDM) supplemented with 10% fetal bovine serum (FBS).

All conditions were incubated for 24 and 48 h. At the end of the incubation periods, the cells were resuspended, washed with PBS, and centrifuged to collect the cell pellet for total RNA extraction.

### 2.6 Cell Culture

The in vitro study utilized the human fibroblast cell line CCD‐1064Sk (ATCC CRL‐2076). Cells were cultured at 37°C in IMDM supplemented with 10% FCS, 1% 200 mM glutamine, 100 IU/mL penicillin, and 100 μg/mL streptomycin, in an incubator with humidified atmosphere at 5% CO_2_. The culture medium was renewed two to three times a week and were allowed to grow at 80% confluence in 75 cm^2^ polystyrene flasks.

Cell viability and cell number estimation were determined using the Trypan Blue dye exclusion test, by counting the unstained cells in the Luna automatic cell counter (Logos Biosystems).

### 2.7 Real‐Time PCR

RNA from the samples was extracted with affinity columns following the instructions of the PureLink RNA MiniKit (Life Technologies). The steps to obtain RNA were as follows: cell lysis, homogenization, binding, washing, and elution. Total RNA concentration was then measured with the NanoDrop ND‐1000 spectrophotometer (ThermoFisher Scientific). Retrotranscription was performed from 9 μL of RNA according to the commercial High‐Capacity RNA‐to‐cDNA kit (Applied Biosystems) and using a thermal cycler MiniAmpPlus Thermal Cycler (Applied Biosystems).

For real‐time PCR, 2 μL of cDNA from each sample was used with TaqMan Fast Advanced Master Mix reagent (Applied Biosystems) and the corresponding gene amplification probes (all TaqMan Gene Expression Assays, Inventoried XS, Applied Biosystems):

Collagen type IV alpha chain 1 (COL4A1, Hs00266237_m1), Collagen type VI alpha chain 1 (COL6A1, Hs01095585_m1), and Elastin (ELN, Hs00355783_m1). The probes for the endogenous controls were beta actin (ACTB, Hs99999903_m1) and pre‐ribosomal 45S RNA 5 (45S5 RNA, Hs03928985_g1). The reactions were performed on a 96‐well MicroAmp Optical reaction plate with barcode (Applied Biosystems) and the equipment used was the QuantStudio 7 Pro (Applied Biosystems).

### 2.8 Statistics

The control condition used as a reference for the analyses was the condition for cells cultured with medium alone. The statistical analysis of the results obtained from the real‐time PCR experiments was performed using the Design and Analysis 2.6.0 software (Applied Biosystems).

## 3 Results

### 3.1 Clinical Results

**FIGURE 2:**
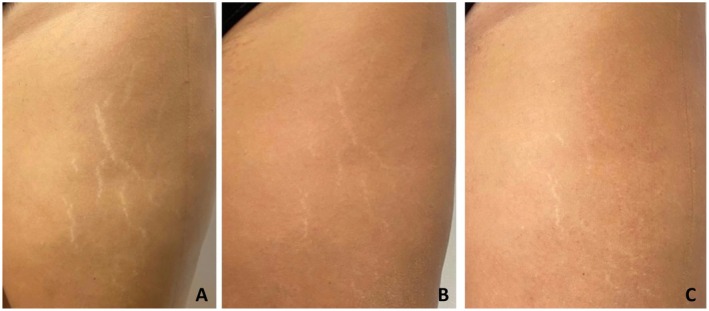
Visual comparison of stretch marks before treatment (A). 1 month after treatment (B), and 6 months after treatment (C). The images illustrate changes in skin appearance following the procedure.

**FIGURE 3:**
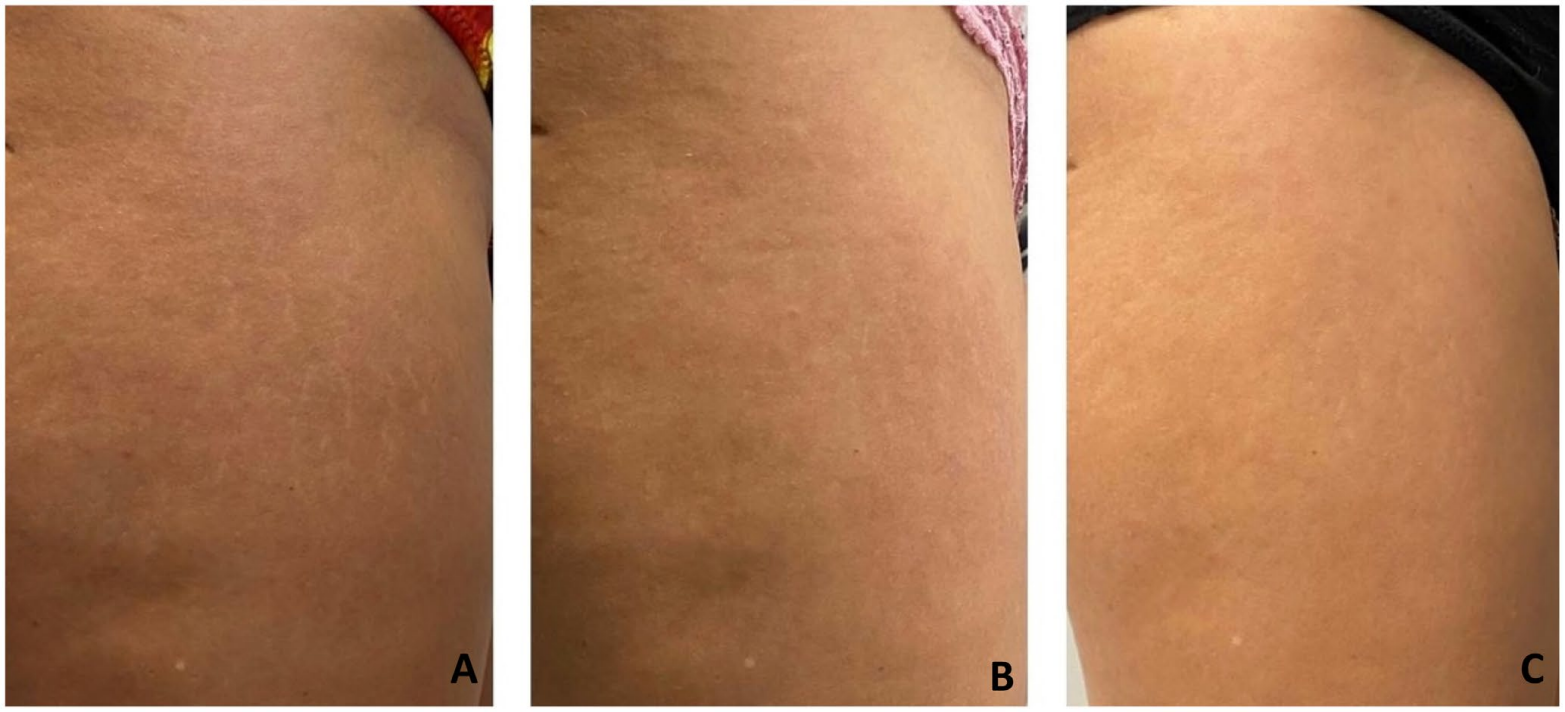
Visual comparison of stretch marks before treatment (A), 1 month after treatment (B), and 6 months after treatment (C). The images illustrate changes in skin appearance following the procedure.

**FIGURE 4:**
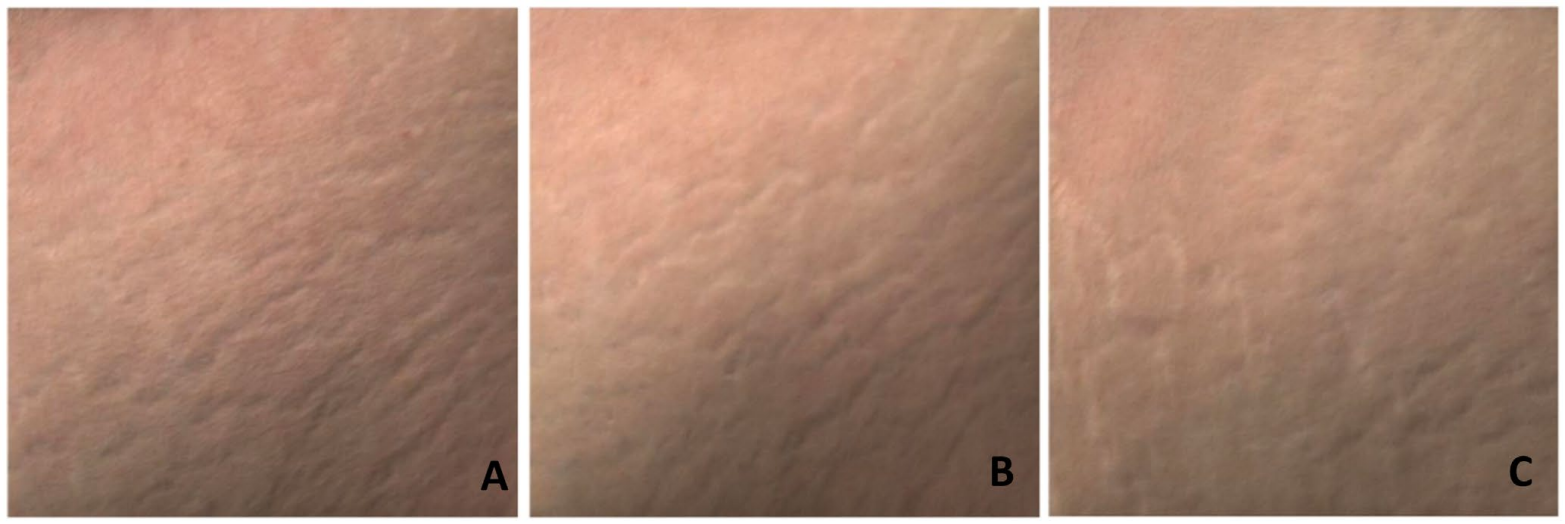
Evaluation of skin texture before treatment (A), 1 month after treatment (B), and 6 months after treatment (C). The images highlight changes in surface smoothness and structural appearance over time.

The clinical outcomes of the treatment were assessed through visual evaluations and photographic documentation taken at various follow‐up points, 1‐ and 6‐month post procedure. The images demonstrate improvements in the appearance of stretch marks in the patients treated with Rigenera Technology, with a visible reduction in their intensity and extent over time, suggesting a positive effect of the treatment. (Figure 2) Comparative photographs taken before the treatment and during follow‐up visits confirm an aesthetic and texture enhancement, which was consistently observed by all the physicians involved in the procedure (Figures 3 and 4).

### 3.2 Patients Satisfaction

Patient satisfaction with the treatment of SD using Rigenera Technology was notably high. No severe adverse effects were registered, and the donor area showed proper healing within the expected physiological timeframes, confirming the safety and tolerability of the procedure. Four of the 10 patients enrolled reported mild and transient symptoms, such as itching and a sensation of skin tension, which persisted for up to one month post‐treatment. These symptoms were manageable and did not significantly impact daily activities. Overall, patients expressed satisfaction with the visible improvements in the appearance of their SD, appreciating the minimally invasive nature of the procedure and the absence of complications.

### 3.3 Collagen and Elastin Expression After Micrograft Suspension Exposure

**FIGURE 5:**
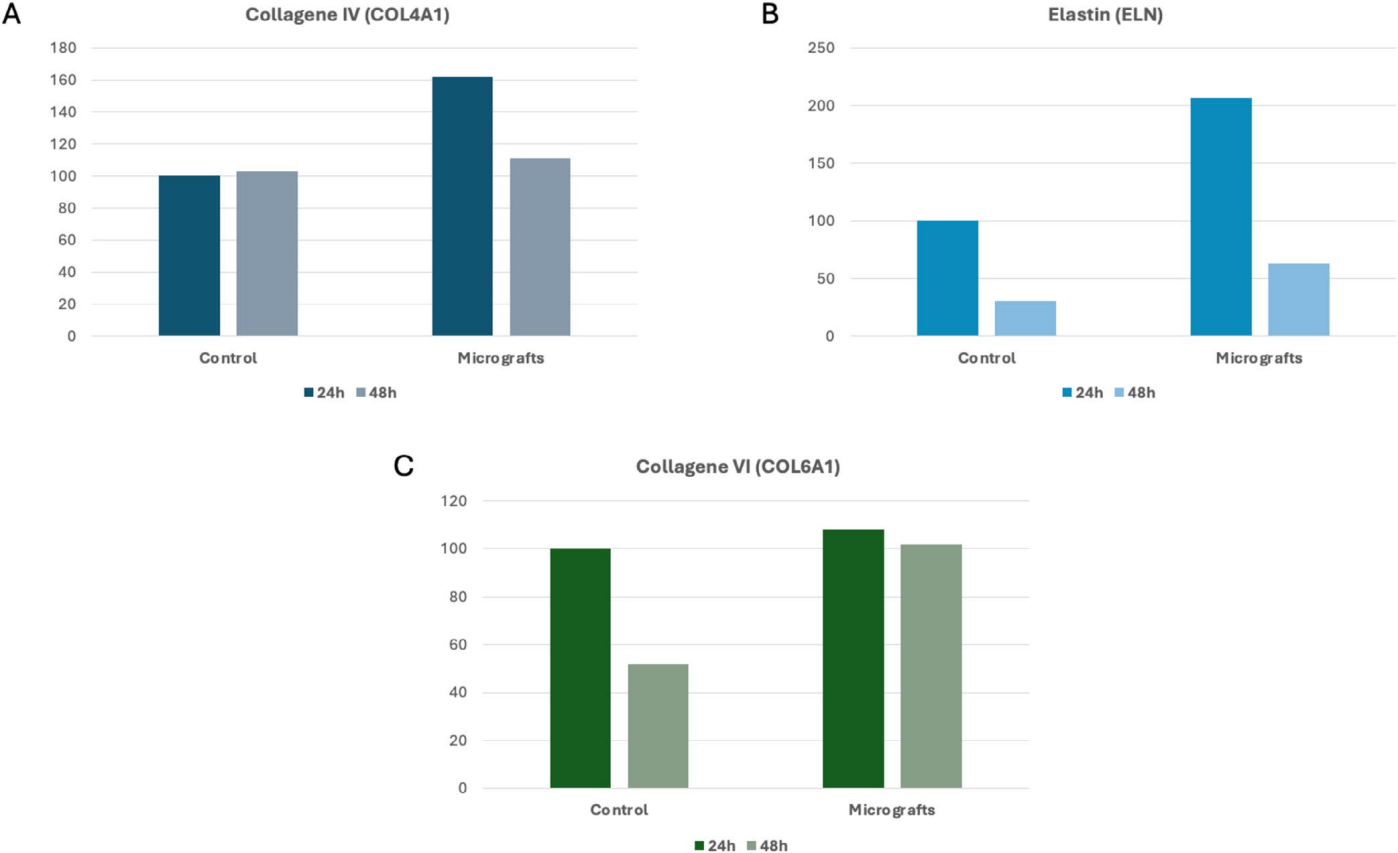
Gene expression results obtained by RT‐PCR in fibroblasts cultured with standard medium (control) or micrograft suspension for 24 and 48 h. Gene expression of (A) Collagen 4 (COL4A1), (B) Elastin (ELN), and (C) Collagen 6 (COL6A1). The data show the effects of micrograft suspension on the expression of key extracellular matrix components over time compared to the control group.

The results of Real Time PCR indicate significant differences in the expression of Collagen types IV (COL4A1) and VI (COL6A1), as well as Elastin (ELN) (Figure 5), between the control group and the cells treated with the micrograft suspension (Rigenera). For COL4A1, cells treated with Rigenera exhibited markedly higher expression levels at both 24 and 48 h of culture compared to the control, with a peak at 24 h. Similarly, COL6A1 showed a comparable trend, where the micrografts‐treated cells maintained higher expression levels at both time points, although the control group demonstrated a notable decrease at 48 h. Regarding ELN, culturing the cells with the micrografts resulted in a high increase in gene expression particularly at 24 h, with values twice as high as in the control group. These results suggest that the micrograft suspension enhances the synthesis of extracellular matrix components, which may contribute to improved tissue regeneration and repair.

## 4 Discussion

The results described above showed that micrografts obtained by Rigenera Technology have a positive outcome in the management of stretch marks. These results are in line with a previous study where it has been demonstrated that micrografts are able to significantly promote skin repair, collagen remodeling, and enhanced tissue elasticity [24].

To confirm the results, Horie et al. [22] conducted a study demonstrating that the application of micrografts in the treatment of stretch marks yielded promising outcomes. Specifically, patients who underwent treatment with the Rigenera protocol exhibited significant improvements in skin texture and roughness, as well as a more uniform skin appearance, suggesting an overall regenerative effect.

Similarly, a more recent study by Girão and Pinto [23] confirmed these findings, highlighting that a single session of micrografts led to substantial enhancements in skin hydration, elasticity, thickness, and microcirculation over a 3‐month period. Additionally, the treatment contributed to a visible reduction in stretch marks, achieved through improved pigmentation and a decrease in skin roughness. According to previous studies, also in this case patients treated with the Rigenera protocol experienced an improvement in skin texture, reduction in roughness and consequent decrease in the size of stretch marks. The procedure was well tolerated by all patients, with no adverse events but only mild symptoms such as itching and tension in the treated area in the weeks following treatment. Stretch marks develop when the elastic fibers within the skin's middle layer, primarily composed of collagen and elastin, rupture due to excessive stretching [25, 26]. These fibers play a crucial role in maintaining skin flexibility and beyond the structural damage, the formation of stretch marks is also influenced by molecular mechanisms [18, 27]. Specifically, key genes responsible for producing collagen and elastin are directly involved in this process, as they regulate the skin's ability to withstand stretching and recover its original shape [28, 29]. When their function is compromised, the skin loses resilience, leading to the characteristic striations associated with stretch marks [30, 31].

To investigate the molecular mechanism underlying the treatment of stretch marks, an in vitro analysis was conducted to evaluate the effects of micrografts when they were placed in culture with fibroblasts. The results demonstrated an upregulation of collagen and elastin gene expression after 24 and 48 h of co‐culture, suggesting that the introduction of autologous micrografts actively stimulates the biological pathways responsible for dermal regeneration or skin remodeling.

These findings align with previous studies highlighting the regenerative potential of autologous micrografts in other dermatology applications [32] further reinforcing their role in skin repair and rejuvenation. The observed increase in collagen and elastin gene expression, two essential components of the dermal extracellular matrix [33], suggests that micrografts actively contribute to the restoration of skin structure by stimulating fibroblast activity and promoting extracellular matrix remodeling. Since the degradation of collagen and elastin is a well‐documented characteristic of stretch mark formation, the ability of micrografts to enhance the production of these structural proteins highlights their potential in mitigating the degenerative effects associated with this condition. By fostering a more favorable microenvironment for cellular repair, they may help restore the mechanical properties of the skin, improving its elasticity and overall appearance.

However, the present study has some limitations given the small sample size of this study, further research involving a larger cohort of patients is essential to validate these findings and enhance the statistical significance of the analysis. Future studies should incorporate long‐term follow‐ups to assess the durability of the observed effects, as well as standardized outcome measures such as histological evaluations, imaging techniques, and patient‐reported assessments to provide a more comprehensive understanding of the clinical benefits.

## 5 Conclusions

In conclusion, this pilot study provides promising preliminary evidence supporting the safety and efficacy of autologous micrografting technology in the treatment of stretch marks. These promising findings underscore the need for further research to validate and expand the clinical applications of this innovative approach, supporting its wider adoption in dermatological practice.

## Author Contributions

Conceptualization: V.P. Methodology: R.T, A.G, R.P, and O.S. Investigation: R.T, A.G, R.P, and O.S. Writing – original draft preparation: V.P. Writing – review and editing: R.T, A.G, R.P, and O.S. Supervision: R.T, A.G, R.P, and O.S. All authors have read and agreed to the published version of the manuscript.

## Ethics Statement

For this study, review and ethical approval was waived as it fell into the category of ‘autologous use in a single surgical step, minimal manipulation, mono‐functional use (used for the same essential function in the recipient and donor) and manipulation with devices under aseptic conditions. Specifically, the medical device used to obtain the autologous micrografts is a CE certified Class II medical device and placed on the market for more than 10 years now. The study was conducted in accordance with the Declaration of Helsinki.

## Consent

Informed consent was obtained from all subjects involved in the study.

## Conflicts of Interest

The authors declare no conflicts of interest.

## Data Availability

The data that support the findings of this study are available from the corresponding author upon reasonable request.
